# Mcl-1 stabilization confers resistance to taxol in human gastric cancer

**DOI:** 10.18632/oncotarget.20222

**Published:** 2017-08-12

**Authors:** Wu Shuang, Lili Hou, Yan Zhu, Qun Li, Wanglai Hu

**Affiliations:** ^1^ Department of Immunology, Anhui Medical University, Hefei, China; ^2^ Department of Clinical Nutriology, The First Affiliated Hospital of Anhui Medical University, Hefei, China; ^3^ Department of Anesthesiology, The First Affiliated Hospital of Anhui Medical University, Hefei, China

**Keywords:** Mcl-1, PI3K/Akt, Taxol-resistance

## Abstract

Taxol has been extensively used as an antineoplastic drug to treat human gastric cancer. However, the acquired drug resistance invariably develops and greatly limits the therapeutic efficacy of Taxol. Identification of the underlying resistance mechanisms may inform the development of new therapies of gastric cancers to Taxol treatment. Here we report that upregulation of Mcl-1 (Myeloid cell leukemia-1) confers acquired resistance to Taxol in human gastric cancer. Mcl-1 is shown to be stabilized in Taxol -resistant gastric cancer cells because of the hyper-activation of the PI3K/Akt signaling pathway. The increased Mcl-1 prevents of the permeabilization of the outer mitochondrial membrane, thereby blocking the Taxol-induced apoptosis. Furthermore, inhibition of Mcl-1 or PI3K/Akt pathway significantly reversed the resistant phenotype of Taxol-resistant human gastric cancer cells. Taken together, our findings broaden the view of PI3K/Akt pathway as an important regulator in Taxol acquired resistance, and implicate Mcl-1 as a specific therapeutic target for the treatment of Taxol-resistant human gastric cancer.

## INTRODUCTION

Gastric cancer, one of the most common malignant tumors in the world, is the third most common cause of cancer mortality worldwide. Patients with gastric cancer are often diagnosed in advanced stages and therefore associated with poor prognosis [1].

The mainstream treatment for the patients with gastric cancer at advanced stage or relapse after surgery is chemotherapy [2].

Taxol is a natural product with potent anti-tumor effect that widely used in gastric cancer therapy. Taxol against gastric cancer mainly through promoting the polymerization of tubulin, and thereby disrupting the normal microtubule dynamics required for cell division and interphase processes. Although the anti-tumor effects of Taxol are commonly exhibited through its induction of mitotic catastrophe, there is compelling evidence suggesting that Taxol acts through different apoptotic mechanisms, such as down-regulates Bcl-2 and Bcl-xL expression, and causes cell cycle arrest at the G2/M phase [3, 4]. Nevertheless, the response to Taxol therapy is limited by acquired resistance.

Over the past years, some mechanisms of acquired resistance to Taxol have been elucidated. Mutations of tubulin were reported to be a strong determinant of Taxol resistance in non-small cell lung cancer [5, 6]. Alterations in tubulin expression and polymerization dynamics are considered to be related to resistance to Taxol [7]. In addition, membrane phosphoglycoproteins that function as drug-efflux pumps were shown to have an important role in the development of resistance to Taxol [8]. Recently, emerging evidence suggests that several pathways, such as the mitochondrial pathway and the EGFR pathway were involved in Taxol resistance. Critical genes in regulation of these pathways have been discovered, e.g., Bcl-2, Bcl-xl and p53. Regulation of these apoptosis-related genes also been involved in the regulation of Taxol acquired resistance [9–13]. Given the diversity and complexity of these identified mechanisms associated with Taxol acquired resistance, fully defining the underlying mechanisms of resistance is still a priority to develop rational strategies to improve the therapeutic efficacy of human gastric cancer cells to Taxol.

To identify novel mechanism(s) of acquired resistance to Taxol, we generated Taxol-resistant cell line by chronic exposure of a human gastric cancer cell line to Taxol. Here we report Mcl-1 as a novel Taxol resistance-associated gene. Mcl-1 is highly upregulated in Taxol-resistant human gastric cancer cells and the up-regulation of Mcl-1 confers the resistance of gastric cancer cells to Taxol. The Mcl-1 is stabilized by hyper-activation of the PI3K/Akt signaling pathway. Increased Mcl-1 is shown to confer Taxol acquired resistance by inhibiting induction of cytochrome C release. We also show that inhibition of Mcl-1 indeed renders gastric cancer cells sensitive to Taxol-induced apoptosis. Collectively, these data suggest that Mcl-1 stabilization response to PI3K/Ak pathway plays an important role in the development of Taxol resistance in human gastric cancer cells.

## RESULTS

### Mcl-1 upregulation confers the resistance of gastric cancer cells to Taxol

**Figure 1:**
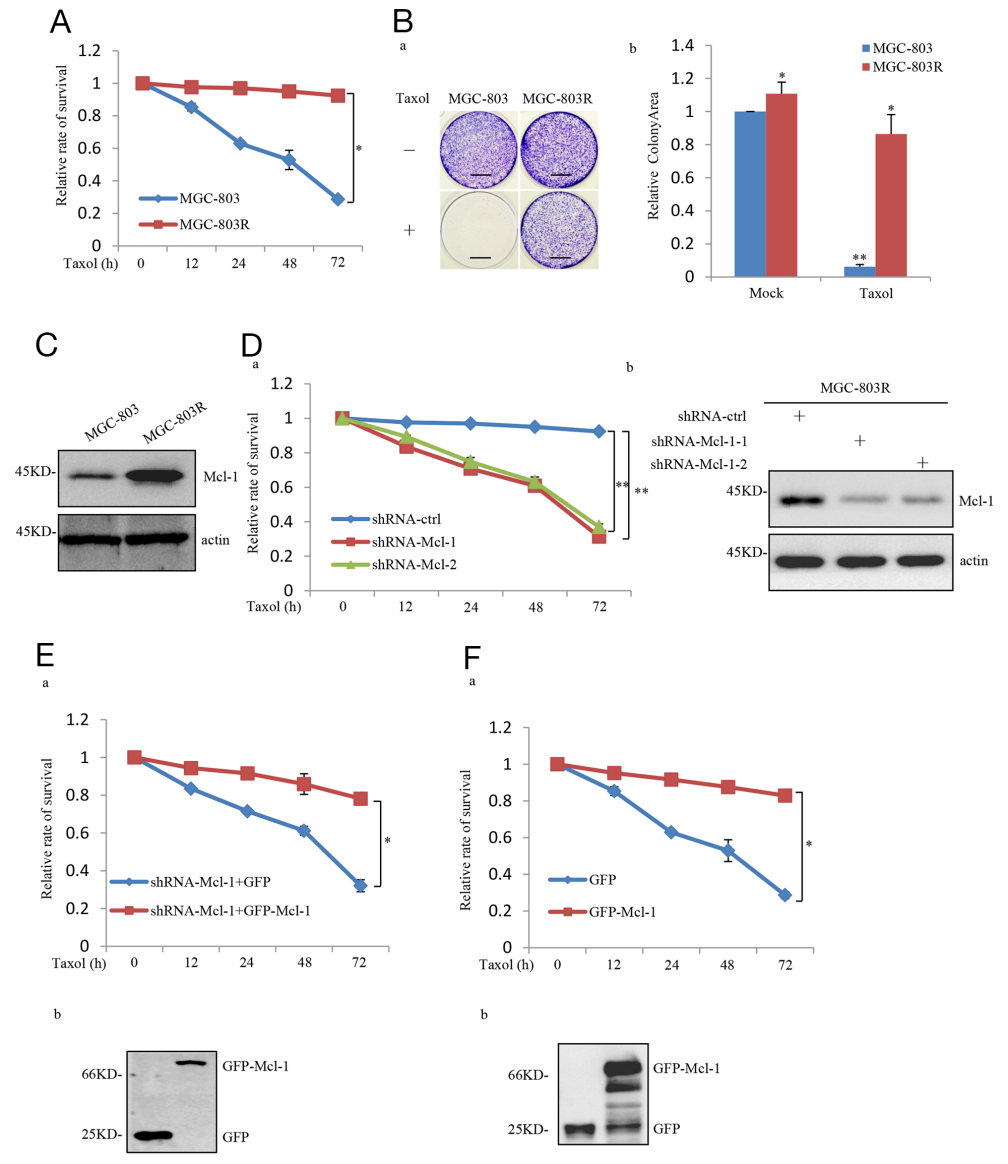
Mcl-1 was identified as a Taxol-resistance associated gene in gastric cancer cell **(A)** MGC-803 and MGC-803R cells were treated with 100 nM Taxol for the indicated periods of time. Viability of cells was determined by the MTT assays. **(B)** Long-term colony formation assay of MGC-803 and MGC-803R cells. Cells were cultured with and without 100 nM Taxol for 3 weeks. Then all dishes were fixed, stained and photographed, Scale bar, 1 cm. (a) and the relative colonyarea was analyzed by image J software (b). **(C)** Proein level of Mcl-1 in MGC-803 and MGC-803R cells was analyzed by western blot analysis. **(D)** MGC-803R cells stably expressing the control shRNA, Mcl-1 shRNA1 or Mcl-1 shRNA 2 were treated with 100 nM Taxol for the indicated periods of time, then the cell viability was examined by the MTT assays (a) and the shRNA-mediated Mcl-1 knockdown was confirmed by western blot analysis (b). **(E)** MGC-803R cells with Mcl-1 stably knockdown were transfected with either GFP or GFP-Mcl-1. After transfection, cells were treated with 100 nM Taxol for the indicated periods of time and then the cell viability was examined by the MTT assay. **(F)** MGC-803 cells stably expressing GFP or GFP-Mcl-1 were treated with 100 nM Taxol for the indicated periods of time. Then cell viability was determined by the MTT assay (a) and the expression of GFP and GFP-Mcl-1 were confirmed by western blot analysis (b).

The development of acquired resistance greatly dropped the efficacy of Taxol in human cancer treatment. To investigate the molecular mechanism(s) responsible for the acquired resistance of gastric cancers to Taxol, we generated a Taxol-resistant cell line by chronic exposure of gastric cancer cell line MGC-803 to 50 nM Taxol for four weeks. This Taxol-resistant cell line was referred to as MGC-803R hereafter. The resistance of MGC-803R was become stable after approximately 6 months of continuous selection and was confirmed by MTT and long-term colony formation assays (Figure 1A and 1B). In order to identify novel gene candidates involving in the resistance to Taxol, we compared the expression level of a panel of genes in MGC-803 and MGC-803R by western blot analysis and real-time RT-PCR analysis (data not shown). Through this analysis, we found the anti-apoptotic protein Mcl-1 was noticeably upregulated in MGC-803R than MGC-803 (Figure 1C), whereas Bcl-2 and Bcl-xl, the other anti-apoptotic members of the Bcl-2 family, showed little, if any, responses to Taxol acquired resistance (Supplementary Figure 2).

We next determined whether increased expression of Mcl-1 confers acquired Taxol- resistant phenotype of MGC-803R. We first knocked down Mcl-1 in these cells, compared with the control, Mcl-1 knockdown MGC-803R showed a dramatic decrease in cell viability in response to Taxol treatment (Figure 1D). Furthermore, this Taxol-induced cell viability inhibition of Mcl-1 knockdown MGC-803R cells was reversed by ectopic expression of Mcl-1 (Figure 1E). We next evaluated this effect of Mcl-1 on Taxol-induced inhibition on cell viability in MGC-803 cells. As shown in Figure 1F, the inhibitory effect was greatly reversed by ectopic expression of Mcl-1 in MGC-803 cells.

To further confirm the effect of Mcl-1 upregulation in acquired Taxol resistance *in vivo*, we performed a xenograft mouse model. MGC-803R cells with and without stable knockdown of Mcl-1 were individually injected subcutaneously into the right flank of nude mice, and mice were treated with Taxol (20mg/kg) through intraperitoneal injection. As shown in Supplementary Figure 4A, MGC-803R xenografts were largely abolished by Mcl-1 knockdown. This was also confirmed by a significant decrease in xenograft weights (Supplementary Figure 4B). Taken together, these results strongly suggest the critical role of Mcl-1 in the acquired resistance to Taxol in human gastric cancer cells.

### Mcl-1 stabilization is regulated by the PI3K/Akt pathway

**Figure 2:**
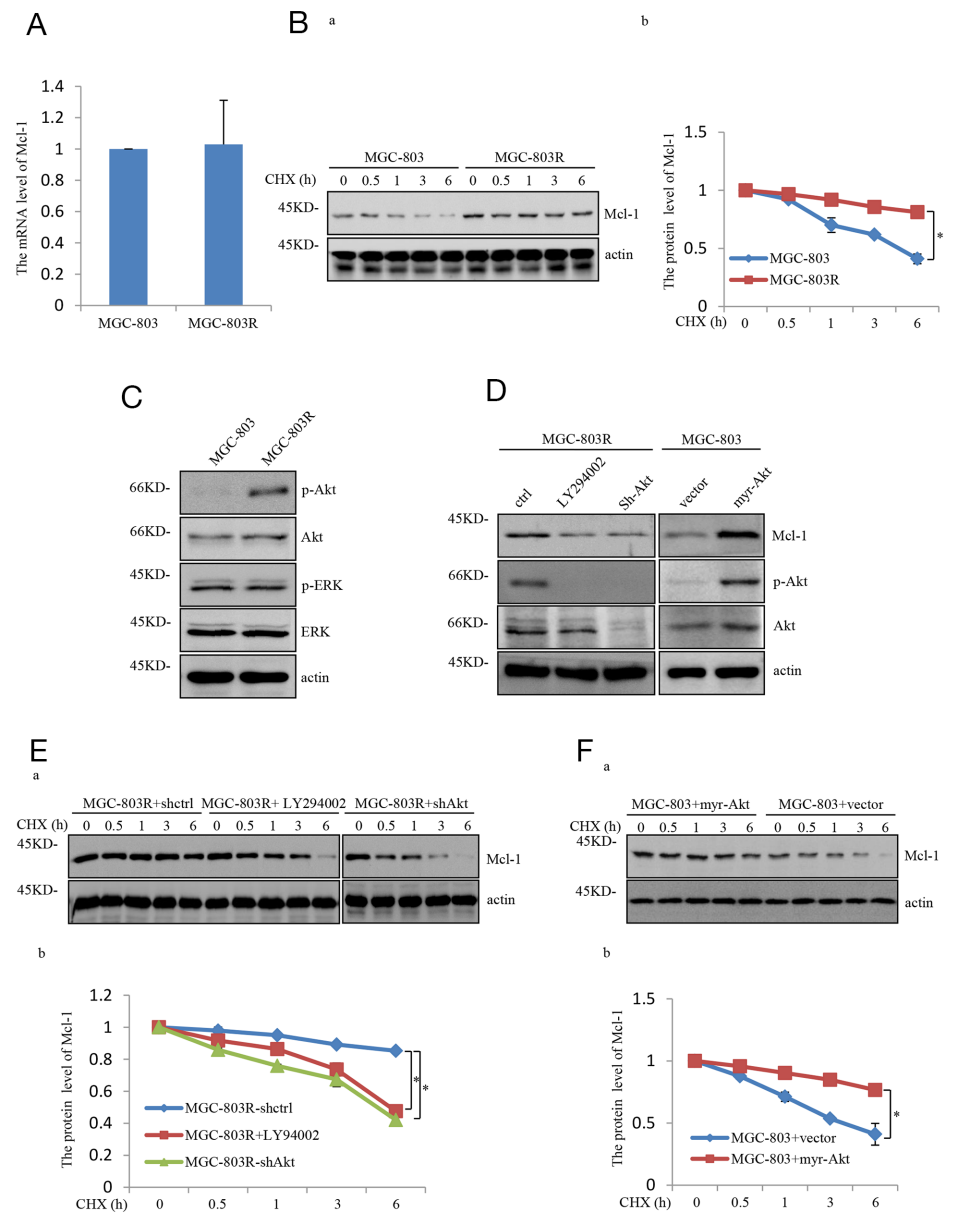
Mcl-1 stabilized responses to PI3K/AKT pathway hyper-activation **(A)** mRNA levels of Mcl-1 in MGC-803 and MGC-803R cells were analyzed by qRT-PCR. **(B)** MGC-803 and MGC-803R cells were treated with cycloheximide (CHX) for the indicated periods of time. Then the cell lysates were analyzed by western blot analysis with the indicated antibodies (a) and the level of Mcl-1 was quantified by Image J (b). **(C)** Lysates from MGC-803 and MGC-803R cells were subjected to western blot analysis with the indicated antibidies. **(D)** MGC-803R cells stably expressing shRNA-ctrl or shRNA-AKT or treated with LY294002 for 12 hours together with MGC-803 cells with and without myr-AKT overexpression. Then cell lysates were subjected to western blot analysis with the indicated antibodies. **(E)** MGC-803R cells with and without AKT stably knockdown together with MGC-803R cells treated with LY294002 for 12 hours were treated with cycloheximide for the indicated periods of time. Cell lysates then were analyzed by western blot analysis (a) and the level of Mcl-1 was quantified by Image J (b). **(F)** MGC-803 cells expressing myr-AKT or vector control were treated with cycloheximide for the indicated times and then cell lysates were evaluated by western blot analysis with the indicated antibodies (a) and the level of Mcl-1 was quantified (b).

We next investigate the molecular mechanism whereby Mcl-1 was upregulated in MGC-803R cells. Mcl-1 mRNA levels were not affected in MGC-803 and MGC-803R cells, as determined by real-time RT-PCR analysis (Figure 2A). However, Mcl-1 half-life was increased in MGC-803R cells (Figure 2B). These data suggest that Mcl-1 was upregulated in MGC-803R cells due to the increased protein stability.

The PI3K/Akt pathway has previously been demonstrated to increase Mcl-1 stability by inhibiting the phosphorylation of Mcl-1 [14]. Intriguingly, MGC-803R cells were shown to exhibit higher levels of phosphorylated Akt (S473)(p-Akt) than MGC-803 cells, but the phosphorylated ERK(p-ERK) showed similar levels in these two cells (Figure 2C), indicating that PI3K/Akt pathway is specifically enhanced in Taxol-resistant gastric cancer cells. Previous studies have shown that the PI3K/Akt pathway could stabilize Mcl-1 by promoting GSK-3α phosphorylation at serine 21 and GSK-3β phosphorylation at serine 9 [15]. To determine the specific role of the PI3K/Akt pathway in mediating Mcl-1 upregulation, phosphorylation of both GSK3α and GSK-3β were analyzed in MGC-803 and MGC-803R cells. As shown in Supplementary Figure 2, MGC-803R cells indeed exhibit higher levels of phosphorylated GSK3α(Ser21) and phosphorylated GSK3β(Ser9) than MGC-803 cells, although the total GSK3α and GSK3β protein showed similar levels in these two cells.

To further determine the role of the PI3K/Akt pathway in mediating Mcl-1 upregulation, MGC-803R cells were treated with LY294002, a specific inhibitor of PI3K, or shAkt respectively, which inhibits the PI3K/Akt pathway. As shown in Figure 2D and Supplementary Figure 1, LY294002 or shAkt treatment led to a dramatic decrease in Mcl-1 protein level. More importantly, ectopic expression of the constitutively active form of AKT (Myr-AKT) in MGC-803 cells led to a strong increase in Mcl-1. We also found that the LY294002 or shAkt treatment reduced the Mcl-1 stability in MGC-803R cells and Mcl-1 half-life was increased in MGC-803 cells with Myr-Akt ectopic expression (Figure 2E and 2F). Together, these data demonstrate that Mcl-1 is stabilized by the PI3K/Akt pathway, through which Mcl-1 contributes to the acquired resistance of human gastric cancer cells to Taxol.

### Inhibition of PI3K/Akt pathway renders gastric cells sensitive to Taxol-induced apoptosis

**Figure 3:**
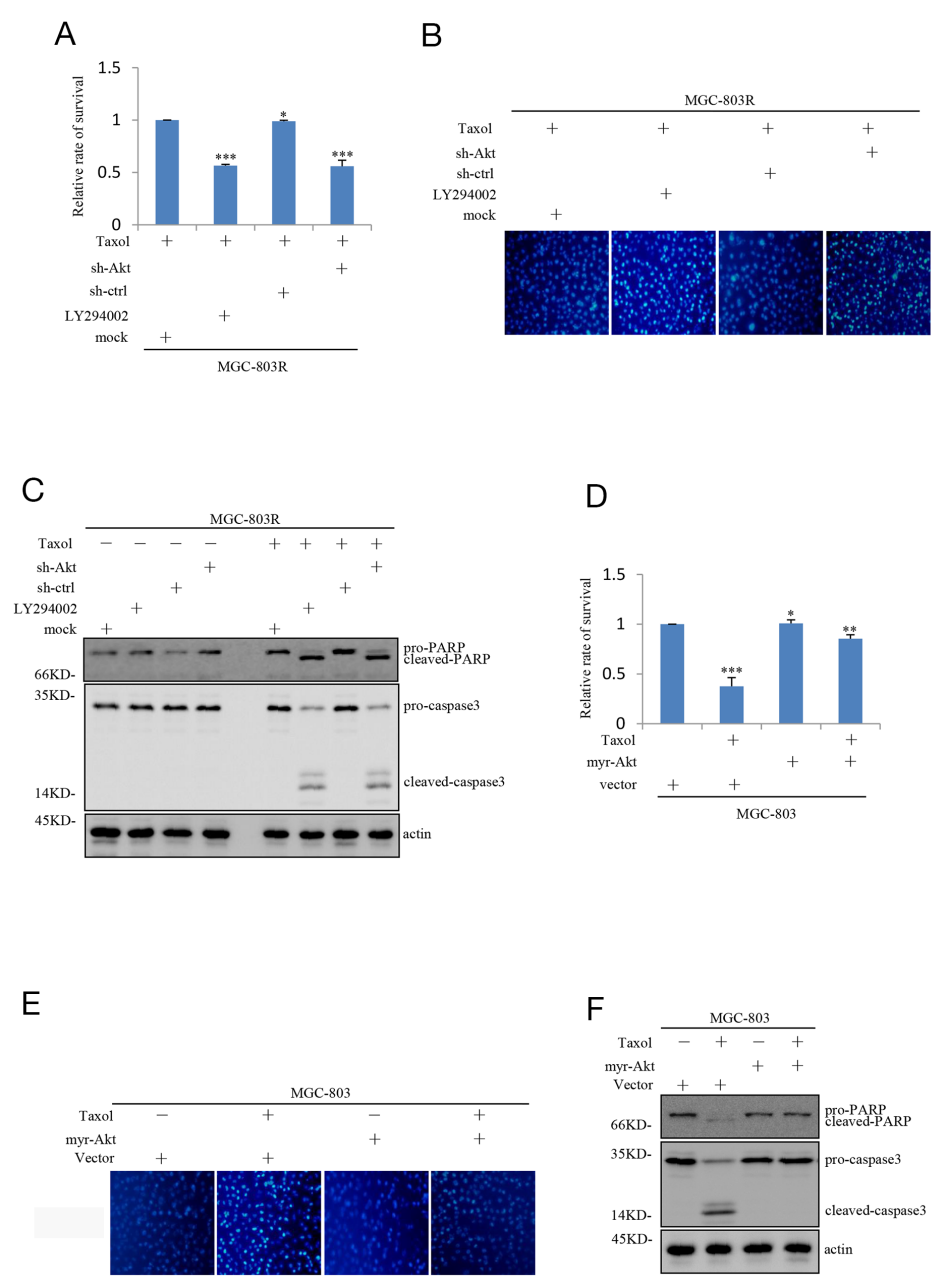
Inhibition of PI3K/AKT pathway attenuates the Taxol-resistance of gastric cells **(A)** MGC-803R cells pre-treated with and without LY294002 for 12 hours together with MGC-803R cells stably expressing shRNA-ctrl or shRNA-AKT were treated with 100 nM Taxol for another 48 hours. Then the cell viability was evaluated by MTT analysis. **(B)** MGC-803R cells pre-treated with LY294002 for 12 hours or stably knockdown AKT were treated with 100 nM Taxol for another 48 hours. Then the cells were stained by Hoechst33342. **(C)** MGC-803R cells pre-treated with LY294002 or stably knockdown AKT were treated with and without 100 nM Taxol for another 48 hours. The cell lysates were then subjected to western blot analysis with the indicated antibidies. **(D)** MGC-803 cells expressing myr-AKT or vector control were treated with and without 100 nM Taxol for another 48 hours. Then the cell viability was determined by MTT analysis. **(E)** MGC-803 cells with and without myr-AKT overexpression were treated with 100 nM Taxol for another 48 hours. The cells were then stained by Hoechst33342. **(F)** MGC-803 cells expressing myr-AKT or vector control were treated with 100 nM Taxol for another 48 hours. Then the cell lysates were subjected to western blot analysis with the indicated antibidies.

We next studied the effect of inhibition of the PI3K/Akt pathway on Taxol-induced apoptosis in Taxol-resistant human gastric cells. More importantly, MGC-803R cells pre-treated with LY294002 or shAkt showed a dramatic decrease in cell viability in response to Taxol treatment (Figure 3A). Photos (Hoechst 33342 fluorescence) documenting the apoptosis was displayed in Figure 3B. Accordingly, MGC-803R cells exhibited high levels of caspase-3 activation and poly(ADP-ribose) polymerase (PARP) cleavage compared with control cells responsive to Taxol treatment under Akt knockdown or PI3K/Akt pathway inhibition (Figure 3C).

We also evaluated the effect of PI3K/Akt pathway on Taxol-induced inhibition on cell viability in MGC-803 cells. As shown in Figure 3D, the inhibitory effect of Taxol on cell viability was greatly reversed by ectopic expression of Myr-Akt in MGC-803 cells. Correlated with this, Myr-Akt overexpression resulted in a great decrease in aberrant nuclei stained by Hoechst 33342 (Figure 3E) and a great decrease in Taxol-induced caspase-3 activation and PARP cleavage (Figure 3F). Take together, these results point out that inhibition of PI3K/Akt pathway can render Taxol-resistant gastric cells sensitive to Taxol-induced apoptosis.

### Inhibiting PI3K/Akt-Mcl-1 axis sensitize gastric cell to Taxol-induced apoptosis is associated with cytochrome C release

**Figure 4:**
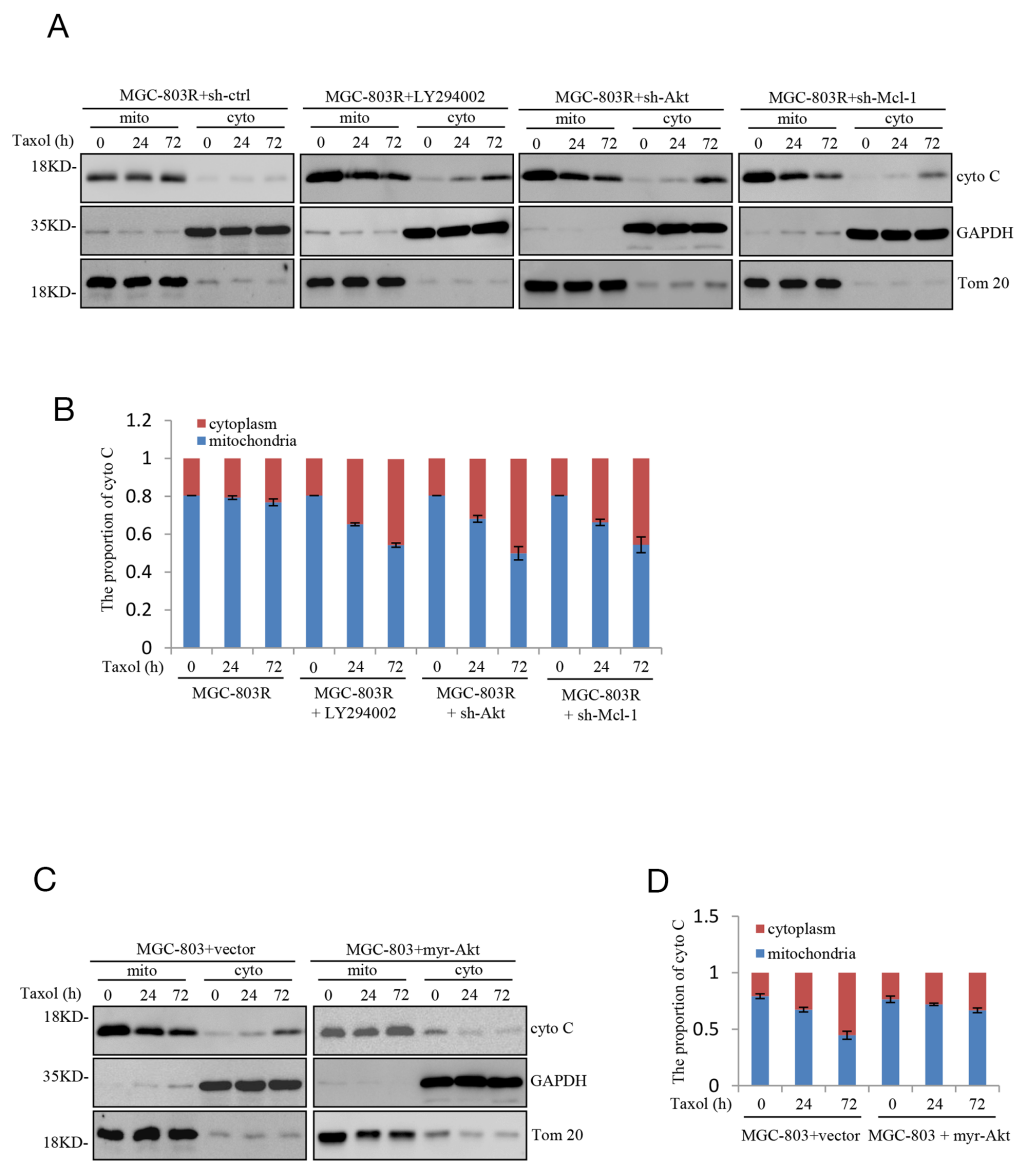
Sensitizes gastric cells to Taxol-induced apoptosis is associated with induction of mitochondrial membrane permeabilization **(A-B)** MGC-803R cells expressing shRNA-AKT, shRNA-Mcl-1 or shRNA-ctrl together with MGC-803R cells pre-treated with LY294002 for 12 hours were treated with 100 nM Taxol for the indicated periods of time. The mitochondrial (mito) and cytoplasmic (cyto) cytochrome C were then isolated and subjected to western blot analysis (A) and the distribution of cytochrome C in mitochondria and cytoplasm was quantified by Image J (B). **(C-D)** MGC-803 cells with and without myr-AKT overexpression were treated with 100 nM Taxol for the indicated periods of time. Then the mitochondrial (mito) and cytoplasmic (cyto) cytochrome C were isolated and subjected to western blot analysis with indicated antibodies (C) and the distribution of cytochrome C in mitochondria and cytoplasm was analyzed by Image J (D).

Next, we explored how PI3K/Akt-Mcl-1 axis contributes to acquired resistance to Taxol in human gastric cells. Mcl-1 has been recognized as an anti-apoptotic regulator of apoptosis by directly or indirectly decrease of the release of the apoptogenic proteins, like cytochrome C [16]. We therefore asked whether PI3K/Akt-Mcl-1 axis could prevent the permeabilization of the outer mitochondria membrane and thus confers resistance to Taxol. Consistent with the previous report, knockdown Mcl-1 indeed cause an increase of the cytosolic expression of cytochrome C in MGC-803R cells upon to Taxol treatment. Furthermore, LY294002 or shAkt treatment which reduces the Mcl-1 expression also induced a dramatic increase of the cytosolic proportion of cytochrome C (Figure 4A and 4B). In contrast, ectopic expression of Myr-Akt in MGC-803 cells markedly inhibited the cytosolic expression of cytochrome C under Taxol treatment (Figure 4C and 4D). Therefore, sensitization of resistant gastric cells to Taxol-induced apoptosis by inhibition of PI3K/Akt-Mcl-1 axis is associated with induction of cytochrome C release.

## DISCUSSION

Chemotherapy for tumor kills target cells primarily by inducing apoptosis. Taxol was obtained from the extract of plant and exhibits potent pro-apoptotic ability in various human malignancies, including gastric cancer. Multiple studies have proved that patients with gastric cancer respond initially to Taxol treatment. Nevertheless, the therapeutic effect of Taxol in treating gastric cancer was limited by the acquired resistance in the majority of patients [17, 18].

Therefore, exploration of the detailed molecular mechanisms of Taxol acquired resistance remains warranted. This would facilitate the development of new therapies and identify novel biomarkers for predicting resistance. In this study, we have provided evidence that increased level of Mcl-1 responses to PI3K/AKT hyper-activation contributes to the development of Taxol acquired resistance.

Mcl-1 is a Bcl-2 family protein and was originally identified as an early induction gene from human myeloid leukemia cell line ML-1 [19]. Subsequent functional studies have shown that Mcl-1 acts as an anti-apoptotic protein and is capable of inhibiting apoptosis induced by various apoptotic stimuli, including etopside, UV irradiation, etc. [20]. Anti-apoptotic protein Mcl-1 protects mitochondrial integrity, thus blocking the release of apoptogenic proteins such as cytochrome C, Smac/DIABLO, and AIF from the mitochondria [16, 21, 22]. More importantly, knockdown of Mcl-1 showed that the anti-apoptotic function of Mcl-1 is essential for maintenance of cell viability [23, 24]. And up-regulation of Mcl-1 is critical for survival of human melanoma cells upon endoplasmic reticulum stress [25]. Although enormous evidences suggest that Mcl-1 be involved in various cellular events, very little is known about the critical function of Mcl-1 in Taxol acquired resistance. Our study therefore uncovers a novel and unexpected function of Mcl-1 in association with Taxol resistance.

In this study, we also provide the detailed mechanism by which Mcl-1 confers acquired resistance to Taxol. Mcl-1 is a short-lived protein with a quick turnover rate due to it contains a PEST (proline, glutamic acid, serine, and threonine) sequence in its Nterminal region [26]. So the post-translational modification is important to maintain cellular protein level of Mcl-1. Mcl-1 is shown to be stabilized responses to PI3K/Akt pathway hyper-activation, thereby confers resistance to Taxol in human gastric cancer cells.

Akt, also referred to as PKB, is activated by various growth and survival factors to function in a pathway involving PI3 kinase [27, 28]. Activated Akt modulates the function of numerous targets involved in the regulation of cell survival and cellular growth. In recent years, it has been shown that the PI3K/Akt pathway is frequently altered in human cancers. Hyper-activated PI3K/Akt pathway mediated the survival of tumor cells and it may decisively contribute to the resistant phenotype [29]. But how PI3K/Akt pathway regulates the Taxol acquired resistance is not fully understood.

As mentioned earlier, several mechanisms of Taxol-resistance have been proposed. In this study, we also validated the effect on knockdown of Taxol-resistance associated genes that have been proposed before, such as TNFAIP1 [30], GBP1 [31] and LIN28 [32]. As shown in Supplementary Figure 3, knockdown of these genes did not significantly alter sensitivity of MGC-803R cells to Taxol-induced apoptosis. These data indicate that upregulation of Mcl-1 represents a unique mechanism of acquired resistance to Taxol.

Taken together, we present an anti-apoptosis mechanism downstream of PI3K/Akt and demonstrate that stabilization of Mcl-1 is a vital consequence of hyper-activation of the PI3K/Akt pathway. Further, we find that the enhanced Mcl-1 prevents of the permeabilization of the outer mitochondrial membrane, and identifies Mcl-1 as a potential therapeutic target for the treatment of Taxol-resistant human gastric cancer. Inhibition of Mcl-1 or PI3K/Akt pathway significantly reversed the resistant phenotype of Taxol-resistant human gastric cancer cells.

## MATERIALS AND METHODS

### Cell culture and reagents

HEK293T and MGC803 cells were maintained in DMEM containing 10% fetal bovine serum (FBS) and 1% penicillin-streptomycin. All cells were cultured in a humidified incubator at 37°C and 5% CO2. Taxol (T7191) and Cycloheximide (c4859) were obtained from Sigma, Taxol was dissolved in DMSO to make up stock solutions of 50 mg/ml. LY294002 (S1737), UO126 (S1901) and Hoechst33342 (C1026) were purchased from Beyotime.

### Generation of taxol-resistant human gastric cancer cell line

MGC-803 cells were treated with 50 nM Taxol for about 5 days, Then the remaining live cells were transferred to the culture medium containing 50 nM Taxol for at least 4 weeks before they were used for the subsequent studies.

### Cytosolic and mitochondrial subcellular fractionation

Cytosolic and mitochondrial subcellular fractionation was carried out as described previously [33]. Briefly, cells were homogenized in homogenization buffer (20 mM HEPES-KOH, pH 7.5, 1.5 mM MgCl2, 10 mM KCl, 1 mM sodium EGTA,1 mM sodium EDTA, 1 mM dithiothreitol, 250 mM sucrose and protease inhibitor cocktail). The homogenates were subjected to centrifugation at 600g for 5 min at 4°C. The supernatant fraction was collected and centrifuged again at 7000g for 10 min at 4°C to obtain cytoplasmic and mitochondrial fractions. The mitochondrial pellet was washed and solubilized in TNC buffer (10 mM Tris acetate, pH 8.0, 5 mM CaCl2, 0.5% NP-40 and cocktail). The protein concentration was determined with a BCA kit before western bot analysis.

### Western blot analysis

Western blot analysis was performed as described previously [34]. The following antibodies were used in this study: anti-AKT (Cell Signaling, 2920S), anti-phospho-ERK(CellSignaling,4370S), anti-phospho-AKT(Ser473) (Epitomics,2118-1), anti-GAPDH(Santa Cruz Biotechnology, SC-32233), PARP(Santa Cruz Biotechnology, SC-8007), anti-caspase-3(Cell Signaling, 9665S), anti-actin(Sigma, A4700), anti-cytochrome C(Santa Cruz Biotechnology, SC-13156), anti-Tom20(Santa Cruz Biotechnology, SC-136211), anti-Mcl-1(Santa Cruz Biotechnology, SC-819), anti-GFP(Sigma, G1544), anti-ERK(Cell Signaling, 4695S), anti-TNFAIP1(proteintech, 60327), anti-LIN28(proteintech,11724), anti-GBP1(proteintech, 15303), anti-Bcl-2(proteintech, 12789), anti-Bxl-xl(proteintech, 26967), anti-GSK3α(Cell Signaling, 9338), anti-phospho-GSK3α(ser21)(Boster, P03152), anti-GSK3β(Cell Signaling, 12456), anti-phospho-GSK3β(ser9)( Cell Signaling, 5558).

### RNA isolation and qRT-PCR

Total RNA was isolated using Trizol reagent (Invitrogen). The assay was performed as previously described [35]. The following primers were used in this study for Mcl-1: F: 5’-GCCAAGGACACAAAGCCAAT-3’, R: 5’-AACTCCACAAACCCATCCCA-3’

### Colony formation assay

MGC803 and MGC803R cells were plated at a density of 3000 cells per well on a six-well plate and were incubated with and without 50 nM Taxol for 3 weeks. Then cells were washed with PBS and fixed with 70% cold methanol for 15 min, followed by staining with 0.005% (m/v) crystal violet for 30 min at room temperature. After gentle wash, the colonies were photographed.

### MTT assay

Cells were seeded in 96-well plates and treated with and without Taxol for the indicated times. 20ul of 5 mg/ml MTT solution was added to each well followed by incubation at 37°C for 4 h. Then cells were incubated with DMSO for additional 10 min at 37°C. The absorbance at 570nm was detected by a microplate reader.

### Hoechst 33342 staining

MGC803 and MGC803R cells were plated at a density of 15000 cells per well on a 24-well plate, followed with the indicated treatments. The viability of cells was evaluated by counting apoptotic cells characteristic of aberrant nuclei staining by Hoechst 33342.

### Lentivirus package and RNA interference

Lentiviral plasmids PLKO.1 and PLKO.1-shMcl-1 or PLKO.1-shAkt were co-transfected with pREV, pGag/Pol and pVSVG into HEK293T cells using Lipo3000. Viral supernatant was collected 48 h post transfection, filtered and added to cells in the presence of 8μg/ml polybrene. The transduced cells were selected by 0.5μg/ml puromycin. Followed are sequences used to knockdown Mcl-1: 5’- GCTGTGTTAAACCTCAGAGTT-3’ and 5’- GCAGAAAGTATCACAGACGTT-3’; Akt: 5’- GCATCGCTTCTTTGCCGGTAT-3’ and 5’- CGCGTGACCATGAACGAGTTT-3’; TNFAIP1: 5’- CCCATGTCTTTCTACCCTAAT-3’ and 5’- GCTGCTGTACAACAGAAGCAA-3’; LIN28: 5’- GCACCAGAGTAAGCTGCACAT-3’ and 5’- ACCTACTTTCGAGAGGAAGAA-3’; GBP1: 5’- CCTCTGTATCAACTCAGGAAA-3’ and 5’- CGGAAATTCTTCCCAAAGAAA-3’.

### Statistical analysis

Statistical analysis was carried out using Microsoft Excel software. Statistical significance was analyzed by Student’s t test and expressed as a p value. p values lower than 0.05 were considered to be statistical significance.* and ** indicate p <0.05 and p <0.01, respectively.

## SUPPLEMENTARY MATERIALS FIGURES


