# Supplementary material for: Mcl-1 stabilization confers resistance to taxol in human gastric cancer
Source: Oncotarget. 2017 Aug 12;8(47):82981–90. doi: 10.18632/oncotarget.20222 (PMC5669943; doi:10.18632/oncotarget.20222)
Supplement: Supplementary file 1 [file oncotarget-08-82981-s001.pdf]

Mcl-1 stabilization confers resistance to taxol in human gastric cancer

SUPPLEMENTARY MATERIALS

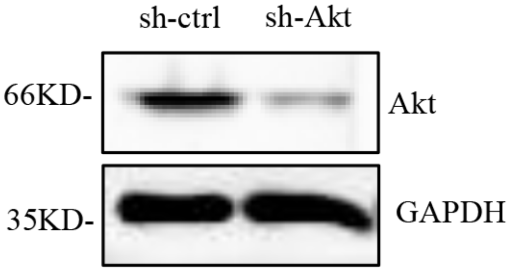

Supplementary Figure 1: The cell lysate of MGC-803R expressing shRNA-AKT or shRNA-ctrl was subjected to western blot analysis with the antibody against Akt.

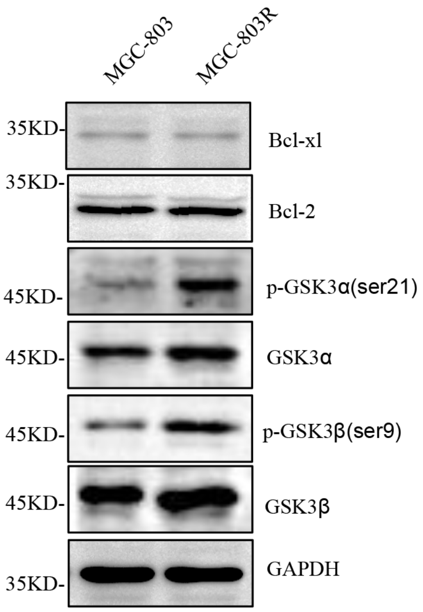

Supplementary Figure 2: Cell lysates of MGC-803 and MGC-803R were evaluated by western blot analysis with the indicated antibodies.

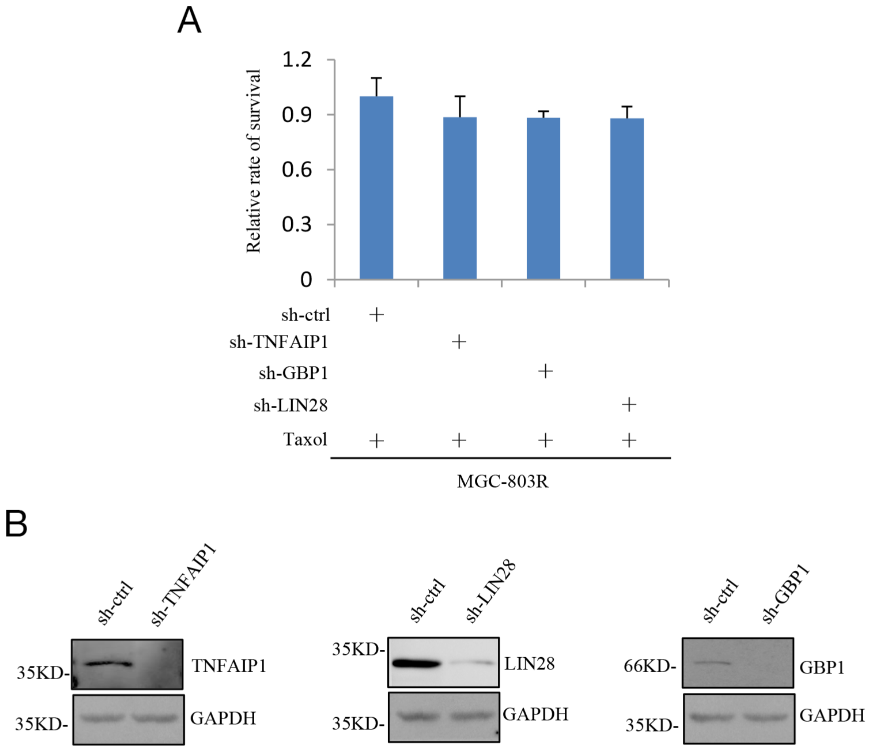

Supplementary Figure 3: MGC-803R cells stably expressing shRNA-ctrl, shRNA-TNFAIP1, shRNA-GBP1 or shRNA-LIN28 were treated with 100 nM Taxol for another 48 hours. The cell viability was evaluated by MTT analysis **(A)** and the knockdown efficiency were evaluated with indicated antibody respectively **(b)**.

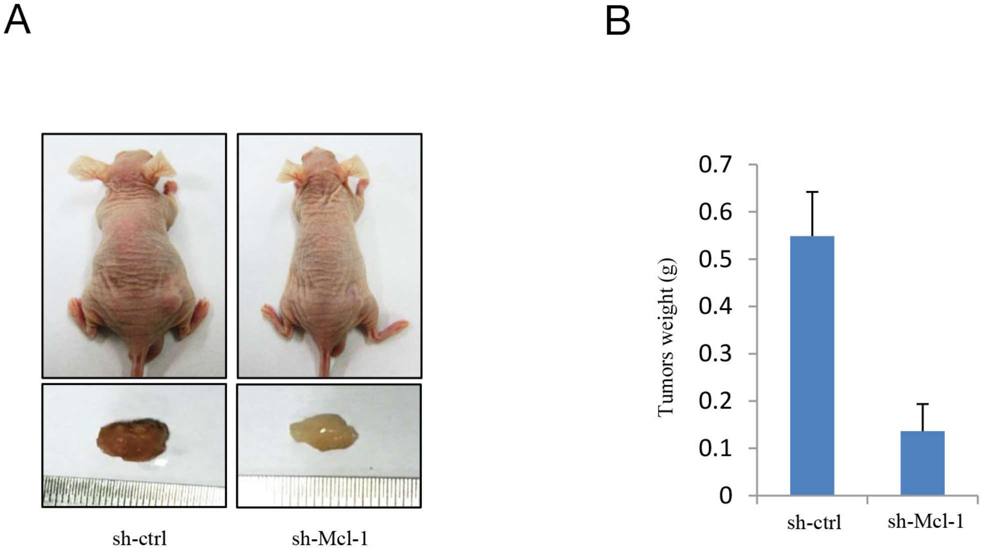

Supplementary Figure 4: MGC-803R cells stably expressing shRNA-ctrl or shRNA-Mcl-1 were individually injected subcutaneously into the right flank of nude mice (n=5) for the tumor formation. And mice were treated with Taxol (20 mg/kg) through intraperitoneal injection. Representative pictures of tumor-bearing nude mice and tumors excised from nude mice were shown **(A)**. Histograms show data representing weights of tumors excised from the indicated mice **(B)**.
